# Effect of Hot-Pressing Temperature on β-Phase Formulation in 3D-Printed Polyvinylidene Fluoride (PVDF)

**DOI:** 10.3390/polym18050617

**Published:** 2026-02-28

**Authors:** Sadia Rahman Toru, Imjoo Jung, Sunhee Lee

**Affiliations:** 1 Department of Fashion and Textiles, Dong-A University, Busan 49315, Republic of Korea; torusadiarahman@gmail.com; 2 Department of Fashion Design, Dong-A University, Busan 49315, Republic of Korea; imjoo629@gmail.com

**Keywords:** polyvinylidene fluoride (PVDF), fused deposition modeling (FDM) 3D printer, hot-pressing, crystallinity, mechanical strength, electrical property

## Abstract

The purpose of this study is to combine 3D printing and hot-pressing to improve polyvinylidene fluoride (PVDF) by making its surface smoother, enhancing crystallinity and electrical and mechanical performance. Before printing, PVDF filament was analyzed using rheology, differential scanning calorimetry (DSC), Thermogravimetric Analysis (TGA), and extrusion tests. Based on these results for printing, 250 °C was fixed as the optimized printing temperature. PVDF samples were printed using an Ultimaker S5 dual-nozzle 3D printer, with a size of 30 × 30 × 0.2 mm^3^. After printing, samples were hot-pressed at five different temperatures, 100, 125, 150, 175, and 200 °C, for 10 min each. Then, the hot-pressed samples were tested using morphology, Fourier transform infrared (FTIR), X-ray diffraction (XRD), DSC, tensile, and electrical properties. From the morphology, the sample thickness decreased from 0.25 to 0.24 mm, making the surface smoother, removing pores after hot-pressing. From FTIR and XRD results, all samples showed similar patterns, but the hot-pressed sample showed slightly stronger β-phase diffraction and peaks near 20° and 840, 1066, and 1275 cm^−1^, indicating better crystal ordering. The DSC results showed a small increase in melting temperature and stable thermal behavior after hot-pressing, confirming improved thermal stability. The tensile property results confirmed that the hot-pressed samples, around 150 and 175, showed higher strength and better flexibility. The electrical I-V test showed stable and uniform conductivity, and the hot-pressed samples performed more consistently. Overall, hot-pressing improved the surface quality, crystallinity, mechanical, and electrical properties of 3D-printed PVDF, making it more reliable for advanced applications.

## 1. Introduction

Polyvinylidene fluoride (PVDF) is a semicrystalline polymer that has been widely used in engineering and functional applications due to its excellent chemical resistance, thermal stability, high mechanical strength, and long-term durability [1,2,3]. Owing to these favorable properties, PVDF has attracted considerable attention for applications in sensors, actuators, energy devices, and other electromechanical systems [2,4,5]. Structurally, PVDF consists of both crystalline and amorphous regions, and its internal microstructure is highly sensitive to processing conditions. Depending on thermal and mechanical history, PVDF can crystallize into several polymorphic phases, including α, β, γ, and δ phases [1,6,7,8,9]. Among these polymorphs, the β-phase is regarded as the most technologically important because its molecular conformation produces a strong net dipole moment. Consequently, β-phase PVDF exhibits superior piezoelectric, pyroelectric, and dielectric properties compared to the non-polar α-phase [6,9,10,11,12,13]. The electroactive β-phase of PVDF, characterized by its all-trans chain conformation, exhibits the highest dipole moment and is primarily responsible for its piezoelectric properties. Enhancement of β-phase content has been widely reported to improve piezoelectric response and energy harvesting performance in PVDF-based systems [14]. For this reason, controlling the crystallinity and phase composition of PVDF is widely recognized as a key factor for enhancing its mechanical and electrical performance, particularly in electromechanical and energy-related applications [11,15,16,17,18]. The crystalline structure of PVDF is highly sensitive to thermal and mechanical processing techniques such as extrusion, stretching, annealing, and hot pressing, where even small variations in temperature, pressure, or deformation can significantly influence phase formation and crystallinity [3,6,9,16,19]. In extrusion-based additive manufacturing methods, including fused deposition modeling (FDM), strong shear forces combined with rapid thermal cycling can induce partial chain alignment and promote β-phase formation during deposition [20,21,22,23]. These microstructural changes directly affect both the mechanical strength and electrical behavior of 3D-printed PVDF components [2,21,24].

In addition, printing parameters such as extrusion temperature, shear rate, printing speed, infill architecture, and cooling conditions play a critical role in determining crystallinity, phase distribution, and electrical anisotropy in 3D-printed PVDF structures [15,21,23,25]. However, due to the layer-by-layer fabrication process, printed PVDF parts often contain interlayer gaps, anisotropic microstructures, and weak interfacial bonding, which can significantly reduce mechanical strength and limit functional reliability [2,11,24,26]. To address these limitations, hot-pressing has been widely employed as an effective post-processing technique to improve the structural integrity and overall performance of PVDF materials [6,8,17,27]. By applying controlled heat and pressure, polymer chain mobility is enhanced; internal porosity is reduced; and denser, more uniform microstructures can be achieved [2,17,27]. Previous studies have demonstrated that optimized hot-pressing conditions can significantly increase the β-phase content and improve the dielectric, ferroelectric, mechanical, and electrical properties of PVDF-based materials [8,17,18,28].

Based on these findings, this study aims to optimize the combined use of 3D printing and hot-pressing as an integrated processing strategy to improve the surface quality, structural uniformity, and crystallinity of PVDF. By carefully controlling printing parameters and hot-pressing conditions, this study aims to refine the internal microstructure of 3D-printed PVDF, thereby reducing defects, enhancing interlayer bonding, and promoting a more uniform crystalline structure. Through this microstructural refinement, the study aimed to improve both the mechanical strength and electrical performance of 3D-printed PVDF, thereby the resulting structural characteristics suggest potential applicability in advanced electromechanical devices, flexible sensors, and piezoelectric or energy-harvesting systems.

## 2. Materials and Methods

### 2.1. Materials

In this study, PVDF filament (3DXTECH, Grand Rapids, MI, USA) was used as the main material for 3D printing. It was provided in a natural form with a measured diameter of 2.85 mm, ensuring stable extrusion during the printing process. The material exhibited a hardness of Shore 98A. Additionally, the filament had a density of 1.71 g/cm^3^, consistent with typical PVDF materials.

### 2.2. Methods

#### 2.2.1. 3D Printing Process of PVDF Samples

**Figure 1:**
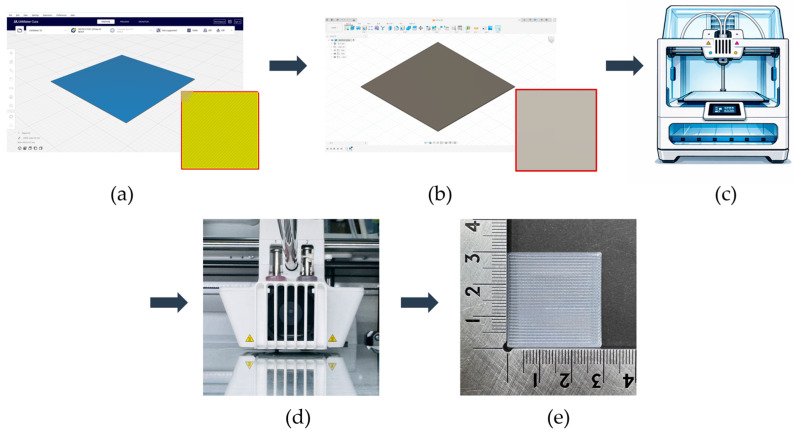
3D printing process of PVDF samples. (**a**) Modeling; (**b**) slicing; (**c**) 3D printer; (**d**) printing; (**e**) 3D-printed PVDF sample.

The PVDF samples were prepared using a 3D modeling, slicing, and printing process. The sample design was created using Fusion (Autodesk Inc., San Francisco, CA, USA) with a size of 30 × 30 × 0.2 mm^3^. After modeling, the design was exported and processed in Ultimaker Cura v5.10.1 (Ultimaker B.V., Utrecht, The Netherlands), where slicing parameters such as layer thickness, infill pattern, and printing orientation were defined. The samples were then printed using a dual-nozzle FDM 3D printer (Ultimaker S5, Utrecht, The Netherlands) equipped with an AA 0.4 mm nozzle. Based on the extrusion test results, the nozzle temperature was set to 250 °C, the bed temperature was maintained at 30 °C, and the printing speed was fixed at 60 mm/s. A gyroid infill pattern with 100% infill density was used to ensure uniformity of the printed samples. These printing conditions enabled smooth extrusion and consistent sample quality. Figure 1 illustrates the 3D printing process of the PVDF samples.

#### 2.2.2. Hot-Pressing Process of 3D-Printed PVDF Samples

**Figure 2:**
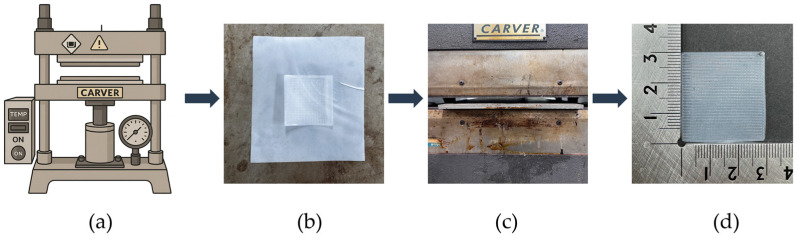
Hot-pressing process of 3D-printed PVDF samples. (**a**) Hydraulic press machine; (**b**) sample preparation; (**c**) sample pressed between stainless steel plate and PTFE film; (**d**) 3DP_PVDF_HP sample.

Figure 2 illustrates the hot-pressing process used on the PVDF samples. The samples were hot-pressed using a hydraulic press machine (12-10H, Carver Inc., Wabash, IN, USA). Each sample was measured 30 × 30 × 0.2 mm^3^. The hot-pressing was carried out at five different temperatures, 100 °C, 125 °C, 150 °C, 175 °C, and 200 °C, and each sample was pressed for 10 min. During pressing, the PVDF sample was placed between two stainless-steel plates, with a size of 200 × 300 × 2 mm^3^. To protect the sample surface and prevent sticking, a polytetrafluoroethylene (PTFE) film (Alphaflon Co., Ltd., Siheung, Republic of Korea) was placed under the sample, and another PTFE film was placed on top before closing the upper plate. The pressure was gradually increased until the upper heated plate came into full contact with the sample and the stainless-steel plates, and the pressing was stopped at this point. The applied load during pressing corresponded to an estimated pressure of approximately 0.13 MPa, calculated based on the sample surface area (30 × 30 mm^2^), and this pressure condition was maintained consistently for all samples. This setup helped the samples press evenly and kept their surfaces clean and smooth without applying excessive pressure. Figure 2 shows the hot-pressing process applied to the 3D-printed PVDF hot-press (3DP_PVDF_HP) samples. After the 10 min pressing time, the pressure was released, and the samples were allowed to cool naturally to room temperature under ambient conditions without external cooling control.

### 2.3. Characterization

#### 2.3.1. Analysis of PVDF Filament

##### Thermal Properties

The thermal properties of the PVDF filament were analyzed using rheology, DSC, and TGA. From the rheological analysis, the thermal–viscoelastic behavior of the PVDF filament was investigated. The measurements were carried out using a rotational rheometer (MCR 102e, Anton Paar, Graz, Austria). The tests were conducted over a temperature range of 40 to 250 °C with a heating rate of 10 °C/min. During the analysis, the loss factor (tan δ) was recorded to evaluate the viscoelastic response of the PVDF filament as a function of temperature. DSC was used to investigate the thermal transitions of the PVDF filament. The measurements were conducted using a DSC 25 instrument (TA Instruments Co., Ltd., New Castle, DE, USA). The analysis was carried out over a temperature range of 30 to 300 °C at a heating rate of 10 °C/min. In total, 3 mg of the filament sample was used for the test. From the DSC curves, the glass transition temperature (T_g_), melting temperature (T_m_), crystallization temperature (T_c_), and enthalpy (ΔH) were analyzed. TGA was conducted to evaluate the thermal stability of the PVDF filament. The measurements were performed using a TGA Q50 instrument (TA Instruments Co., Ltd., New Castle, DE, USA). The analysis was conducted over a temperature range of 30 to 800 °C at a heating rate of 10 °C/min. In total, 3 mg of the filament sample was used for the test to analyze the weight change as a function of temperature.

##### Extrusion Test

To find the optimal printing condition before actual printing, extrusion tests were carried out in which the nozzle temperature was evaluated at six different levels, from 220, 230, 240, 250, and 260 °C. The morphology, weight, and thickness of the extruded lines were examined for each temperature.

#### 2.3.2. Analysis of 3DP_PVDF_HP Samples

##### Actual Weight and Thickness

The actual weight of the 3DP_PVDF_HP was measured using an electronic balance (PAG114, Ohaus Corp., Parsippany, NJ, USA). Both raw and hot-pressed samples were evaluated. For each sample, three specimens were prepared. Each specimen was weighed three times, and the average weight was used for the analysis.

Thickness measurements of the raw and 3DP_PVDF_HP samples were performed using a digital caliper (Absolute Digimatic Caliper, Mitutoyo Corp., Kawasaki, Japan) with a resolution of 0.01 mm and an accuracy of ±0.02 mm, within the 0–100 mm measurement range. For each sample, three specimens were prepared. Each of the samples was weighed three times, and the average weight was used for the analysis.

##### Morphology

The surface morphology of the 3DP_PVDF_HP samples was observed using an optical microscope (NTZ-6000, Nextecvision Co., Ltd., Anyang, Republic of Korea) at a magnification of ×19.5. Images were taken from both the front and back sides of the samples to examine their surface features.

##### Crystalline Property

The crystalline properties of the 3DP_PVDF_HP samples were analyzed using Fourier transform infrared (FTIR) and X-ray diffraction (XRD).

FTIR analysis was carried out on the 3DP_PVDF_HP samples using a FTIR spectrometer (Nicolet Nexus; PerkinElmer, Waltham, MA, USA) with a scan range of 600–4000 cm^−1^. Both raw and hot-pressed samples were analyzed. For each raw and hot-pressed temperature, three samples were prepared, and each sample was measured three times. The average value was used for further analysis. To quantitatively evaluate the β-phase content, the relative β-phase fraction (F(β)) was calculated using the Beer–Lambert-based method proposed by Gregorio and Cestari [29], according to

(1) $$F(\beta )=\left[\frac{A_{840}}{{(K}_{\beta }/K_{\alpha })A_{766}+A_{840}}\;\right]$$

where *A*_840_ and *A*_766_ are the absorbance values at 840 and 766 cm^−1^, respectively, and *K*_β_ (7.7 × 10^4^ cm^2^/mol) and *K*_α_ (6.1 × 10^4^ cm^2^/mol) are the absorption coefficients of the β-phase and α-phase. The absorbance values were calculated from transmittance data according to the Beer–Lambert law, where A = −log10(T/100), with T representing percent transmittance.

XRD analysis was performed using an X-ray diffractometer (Empyrean, PANalytical B.V., Almelo, The Netherlands). Both raw and hot-pressed samples were examined. For each raw and hot-pressed temperature, three samples were prepared. Each sample was scanned twice at two orientations, 0° and 90°, based on the nozzle printing direction. The average diffraction results were used for analysis.

##### Differential Scanning Calorimetry (DSC)

DSC analysis was performed to investigate the thermal properties of the 3D-printed PVDF raw and 3DP_PVDF_HP samples. The measurements were conducted using a DSC 25 instrument (TA Instruments Co., Ltd., New Castle, DE, USA). The analysis was carried out over a temperature range of 30 to 300 °C at a heating rate of 10 °C/min. In total, 3 mg of each sample was used for the test. From the DSC curves, the glass transition temperature (T_g_), melting temperature (T_m_), crystallization temperature (T_c_), and enthalpy (ΔH) were analyzed. To quantitatively evaluate the crystalline development, the degree of crystallinity (*X_c_*) was calculated using the following equation:

(2) $$\chi_{c}(\%)=\frac{\Delta H_{m}}{\Delta H_{m0}}\times 100$$

where $\Delta H_{m}$ represents the measured enthalpy value and $\Delta H_{m0}$ (104.7 J/g) represents the melting enthalpy of 100% crystalline PVDF [29,30].

##### Tensile Property

Tensile testing was conducted to evaluate the mechanical response of the 3DP_PVDF_HP samples based on ISO 527-3 under controlled laboratory conditions [31]. The measurements were performed using a universal testing machine (AGS-X, Shimadzu Corp., Kyoto, Japan). The 3DP_PVDF_HP samples were prepared with ISO 527-3 Type 1B, and a gauge length of 50 mm was applied for all tests. For each hot-pressed condition, three specimens were prepared and tested, and the average values were calculated. During testing, stress-strain curves were obtained and analyzed to extract key mechanical parameters, including initial modulus, maximum stress, strain at break, and toughness.

##### Electrical Property

The electrical property of the 3DP_PVDF_HP samples was evaluated using a source measure unit (2450 Source Meter, Keithley, Tektronix Inc., Beaverton, OR, USA). All measurements were carried out under controlled laboratory conditions to ensure consistency. The 3DP_PVDF_HP samples were tested individually. During the measurements, a voltage range from −1 V to 50 V was applied in 0.1 V intervals, and the corresponding current response was recorded. For each hot-pressed condition, three specimens were prepared and tested, and the average values were calculated based on the electrical measurements. To enable direct comparison among samples with different baseline currents, the electrical response was expressed as a normalized current change (*I_c_*), according to the following equation.

(3) $$I_{c}=\left[\frac{I-I_{0}}{I_{50}}\;\right]\times 100\%$$

where I is the current measured at a given voltage, *I*_0_ is the value of initial current, and *I*_50_ is the value of current at 50 V. The current is measured at the maximum applied voltage of 50 V. In this study, the response was normalized by *I*_50_, corresponding to the current at the maximum applied voltage, to compare voltage-dependent electrical properties among samples.

## 3. Results and Discussion

### 3.1. Thermal Property of PVDF Filaments

**Figure 3:**
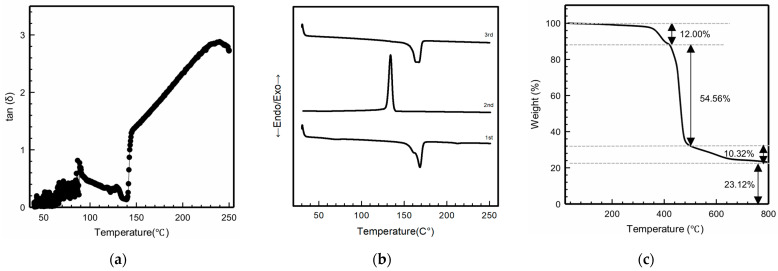
Thermal properties of PVDF filament. (**a**) Rheology; (**b**) DSC thermogram; (**c**) TGA.

Figure 3 presents the results for the thermal properties of the PVDF filament, as analyzed from rheology, DSC, and TGA. As shown in Figure 3a, the rheological results indicated that the loss factor (tan δ) of the PVDF filament exhibited a clear temperature dependence. Below 130 °C, tan δ remained low and showed only small variations. A minor peak appeared near 100 °C, followed by a slight decrease in the range of 130–140 °C. With further increase in temperature, tan δ began to rise gradually above 150 °C and exceeded 1 at approximately 155–160 °C. At higher temperatures between 240 and 250 °C, tan δ increased sharply and reached values of about 2.5 to nearly 3. This temperature-dependent behavior indicated a transition from solid-like to flow-dominated viscoelastic response with increasing temperature, reflecting enhanced polymer chain mobility during thermal processing of PVDF [30].

In Figure 3b, the DSC results of the PVDF filament displayed clear thermal transitions during repeated heating and cooling cycles. Heating and cooling cycles were repeated to distinguish the intrinsic material behavior from prior thermal effects. The first heating shows the as-received state, and the second heating shows the behavior after thermal history is removed. In the first heating run, a glass transition temperature (T_g_) was observed at 69.23 °C, followed by a strong melting peak at 168.42 °C with an enthalpy of 35.33 J/g. During the second run, a crystallization peak (T_c_) appeared at 134.03 °C with an enthalpy of 38.57 J/g. In the third heating run, the melting temperature (T_m_) remained nearly 30unchanged at 167.50 °C with an enthalpy of 35.33 J/g. The repeated heating and cooling cycles showed stable thermal transitions, indicating preserved crystallinity and thermal stability of the PVDF filament, which was consistent with the typical DSC behavior of semicrystalline PVDF materials [30,32,33].

Figure 3c shows the TGA curve of the PVDF filament and exhibits three main stages of weight loss. The initial noticeable weight loss of 12% occurred between 360 and 420 °C, corresponding to the onset of the primary thermal degradation stage of PVDF [30]. The largest weight loss, about 54.56%, took place in the temperature range of 400–500 °C. At higher temperatures between 500 and 600 °C, an additional weight loss of 10.32% was observed, and a residue of about 23.12% remained after heating to 800 °C. These results indicated that the major thermal degradation of PVDF occurred at temperatures far above the typical 3D printing range, confirming sufficient thermal stability of the PVDF filament during processing and subsequent thermal treatments [32,33,34]. Although minor processing-related components may be present, the dominant degradation behavior remains characteristic of PVDF.

Overall, these results showed that the PVDF filament had stable thermal and processing behavior within the tested temperature range. The increase in tan δ with temperature indicated enhanced chain mobility and controllable melt behavior [27], supporting smooth extrusion during the 3D printing process, as it is necessary to ensure uniform filament deposition, stable layer bonding, and consistent print quality without defects, such as nozzle clogging, uneven printing layers, or irregular flow. The consistent melting behavior observed in DSC confirmed stable crystallinity after thermal cycling and showed a clear relationship between PVDF thermal transitions and printing conditions, where T_g_ was related to bed temperature, and T_m_ was associated with nozzle temperature for stable flow and extrusion quality. In addition, the TGA results demonstrated that PVDF had sufficient thermal stability for the 3D printing process and necessary thermal processing [30,32,33,34].

### 3.2. Extrusion Test of PVDF Filaments

Table 1 presents the results of the extrusion test conducted to determine the suitable printing temperature for the PVDF filament. Based on the thermal property analyses, which showed temperature-dependent chain mobility, stable melting behavior, and sufficient thermal stability, the nozzle temperature was set to different temperatures from 220 to 260 °C. The thickness, weight, and surface morphology of the extruded PVDF filament lines were evaluated at each temperature. At 220 and 230 °C, the extruded lines showed a lower thickness of 0.53 ± 0.02 and 0.52 ± 0.01 mm, with weights of 2.70 ± 0.01 and 2.69 ± 0.01 mg. In this temperature range, the extrusion flow was less uniform, which was connected with the rheological results indicating low tan δ values and limited polymer chain mobility. The optimization approach integrates rheological trends with direct extrusion performance evaluation, thereby reflecting the actual flow conditions encountered during fused deposition modeling.

At 240 °C, both thickness and weight increased. However, surface irregularities and bubble formation became noticeable. This behavior corresponded to the onset of increased molecular mobility observed in the rheological analysis, where tan δ began to increase, but stable flow was not fully achieved. At 250 °C, the extruded filament line showed a thickness of 0.60 ± 0.01 mm and a weight of 2.90 ± 0.01 mg. The surface morphology was smooth and continuous, indicating stable and uniform material flow. This result was consistent with the rheological data, which showed flow-dominated behavior, and with the DSC results, which confirmed stable melting and preserved crystallinity. At 260 °C, although the weight remained similar, a slight reduction in thickness was observed. While TGA results confirmed sufficient thermal stability at this temperature, the extrusion behavior showed reduced dimensional stability.

Based on the extrusion results and their relationship with thermal property analyses, 250 °C was selected as the optimal printing temperature for the PVDF filament, as it provided the most stable extrusion behavior and smooth material flow.

### 3.3. Morphology of 3DP_PVDF_HP Samples

Table 2 presents the morphological changes of the PVDF samples before and after hot pressing at different temperatures, at 100°, 125°, 150°, 175°, and 200°. The weight of all samples remained constant at 0.26 ± 0.00 g, indicating that no mass change occurred during the hot-pressing process. On the other hand, the thickness of the 3D-printed samples gradually decreased with increasing hot-pressing temperature. The raw PVDF sample showed a thickness of 0.25 ± 0.00 mm, while 3DP_PVDF_HP samples showed reduced thickness values ranging from 0.22 ± 0.00 mm at 100 and 125 °C, 0.21 ± 0.00 mm at 150 and 175 °C, to 0.19 ± 0.00 mm at 200 °C. As a result, the ratio of decreased thickness increased from 10.67 ± 2.31% at 100 °C to 24.00 ± 0.00% at 200 °C. The surface morphology images of the samples, taken from both the front and back sides, show that the layer structure became more compact and uniform after hot-pressing compared to the raw sample, especially at higher temperatures. The morphology images qualitatively indicate a reduction in interlayer gaps and internal voids after hot-pressing. In addition, the observed decrease in thickness without measurable mass loss suggests improved densification and reduced porosity. These results indicated that hot-pressing effectively densified the 3DP_PVDF_HP samples by reducing thickness while maintaining weight, which was related to improved structural uniformity.

### 3.4. Crystalline Properties of 3DP_PVDF_HP Samples

**Figure 4:**
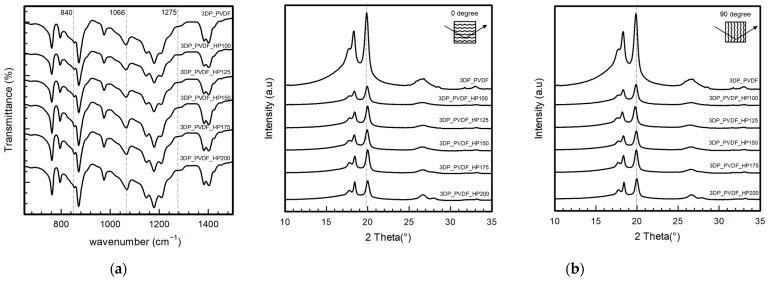
Crystalline properties of 3DP_PVDF_HP samples. (**a**) FTIR; (**b**) XRD in two printing directions.

Figure 4a shows an FTIR graph that displays the spectra of PVDF samples, both raw and after hot-pressing at various temperatures: 100, 125, 150, 175, and 200 °C. All samples had similar main peaks at 840 cm^−1^, 1066 cm^−1^, and 1275 cm^−1^, which were the PVDF’s beta (β) crystalline phases. However, the 3DP_PVDF_HP samples showed slightly sharper and stronger peaks compared to the raw sample. An absorption band near 766 cm^−1^, associated with the non-polar α-phase, was also observed. Although the overall spectral patterns remained similar after hot-pressing, slight changes in peak intensity and sharpness were detected. The calculated β-phase fractions were 41.7% for the raw PVDF sample and 41.9%, 39.9%, 38.7%, 37.2%, and 42.6% for the samples hot-pressed at 100, 125, 150, 175, and 200 °C, respectively. The results show that the β-phase fraction remained within a range of approximately 37–43% across all processing conditions. This indicates that hot-pressing preserved the dominant β-phase structure while enhancing overall crystallinity and structural ordering. Although the overall spectral patterns remained similar, the hot-pressed samples showed more defined β-phase bands, reflecting improved structural ordering. This observation agrees with previous reports on thermally treated PVDF systems [35].

As shown in Figure 4b from the diffraction patterns, it was observed that both the PVDF and 3DP_PVDF_HP samples, which were hot-pressed at 100, 125, 150, 175, and 200 °C, exhibited a clear diffraction peak near 2θ = 20°. This peak was characteristic of the electroactive β-phase of PVDF [30]. Compared to the raw sample, the 3DP_PVDF_HP exhibited sharper and more regular diffraction peaks, indicating improved crystalline ordering and structural uniformity. For both the raw and hot-pressed samples, similar diffraction patterns were observed at nozzle printing orientations of 0° and 90°. The comparable peak positions and intensities indicate that no significant crystallographic orientation or phase alignment differences were introduced by the printing direction. This suggests that hot-pressing contributes to structural uniformity and reduces orientation-dependent crystalline behavior in the printed PVDF samples [30]. The XRD results indicated that hot-pressing enhanced the crystalline quality and structural uniformity of PVDF while preserving its dominant β-phase structure, which was consistent with previous XRD analyses of PVDF [29].

Overall, the FTIR and XRD results showed that hot-pressing could sharpen FTIR and XRD peaks, with improved crystalline quality and structural uniformity without changing the phase composition, which was similar to previous studies reporting enhanced β-phase development and improved crystallinity in thermally and pressure-treated PVDF systems [22,29,30,35,36].

### 3.5. Thermal Properties of 3DP_PVDF_HP Samples

**Figure 5:**
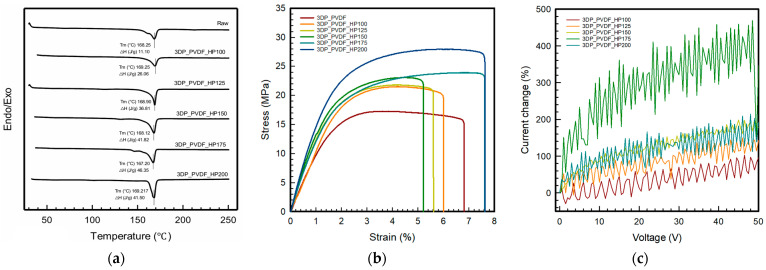
Thermal, mechanical, and electrical properties of 3DP_PVDF_HP samples. (**a**) DSC; (**b**) tensile properties; (**c**) electrical properties.

As shown in Figure 5a, the DSC results show the melting behavior of the raw and 3DP_PVDF_HP samples when heated from 50 °C to 250 °C. For the 3DP_PVDF_HP samples, the first heating cycle was used to evaluate the as-processed state in order to preserve the thermal history induced by printing and hot-pressing. All samples showed similar T_m_ values between 167.20 °C and 169.25 °C. The sample hot-pressed at 100 °C showed a T_m_ of 169.25 °C with an enthalpy of 26.06 J/g, and the sample hot-pressed at 125 °C showed a T_m_ of 168.90 °C and a higher enthalpy of 36.81 J/g. As the hot-pressing temperature increased, the enthalpy further increased to 41.82 J/g at 150 °C, where T_m_ was 168.12 °C, and reached the highest value of T_m_ of 167.20 °C, with an enthalpy of 46.35 J/g at 175 °C. At 200 °C, the T_m_ slightly increases to 169.17 °C, while the enthalpy decreases to 41.50 J/g. The degree of crystallinity (X_c_) increased from 24.9% at 100 °C to 35.2% at 125 °C, 39.9% at 150 °C, and reached a maximum of 44.3% at 175 °C. At 200 °C, X_c_ slightly decreased to 39.6%. The slight decrease in crystallinity at 200 °C may be attributed to partial melting–recrystallization processes [12,30]. At elevated temperatures, enhanced chain mobility can promote lamellar thickening and crystal coarsening, potentially resulting in the formation of larger but fewer crystalline domains [12,22]. This process may reduce overall nucleation density and lead to a slight reduction in total crystalline fraction despite improved crystal perfection. These results demonstrate that moderate hot-pressing temperatures significantly promote crystalline development and structural densification. Although the melting temperatures remain similar, the hot-pressed samples showed smoother and more stable melting peaks, indicating better thermal uniformity and improved crystalline structure compared to the raw sample. Thus, the DSC results showed that hot-pressing improves thermal uniformity and enhances the crystalline development of PVDF without noticeable changes in its melting temperatures. Similar behavior has been reported in previous studies, which showed that hot-pressing and pressure-assisted processing increased the melting enthalpy and crystalline development of PVDF without significant changes in melting temperature [6]. In addition, stable melting temperatures with improved thermal uniformity have been observed in thermally processed and 3D-printed PVDF materials [37].

### 3.6. Mechanical Properties of 3DP_PVDF_HP Samples

Figure 5b presents the tensile results for the 3DP_PVDF_HP samples after being hot-pressed at 100, 125, 150, 175, and 200 °C, and the detailed tensile properties are summarized in Table 3. The stiffness of the samples improved steadily with increasing temperature. The initial modulus increased from 785.08 ± 4.84 MPa in the raw sample to 1046.48 ± 116.36 MPa at 200 °C. Even at 100 °C, the modulus increased to 937.67 ± 10.31 MPa, and a gradual rise was maintained as the temperature increased further. The maximum stress followed a similar tendency. The raw sample exhibited a tensile strength of 18.37 ± 0.96 MPa. After hot-pressing at 100 °C, the strength increased to 21.64 ± 0.55 MPa, and it reached 28.72 ± 4.55 MPa at 200 °C. This trend corresponds to the enhanced beta phase formation and the development of a more ordered crystalline structure at higher hot-pressing temperatures. The strain at break showed a distinct change at higher temperatures. Up to 150 °C, the breaking strain remained within a narrow range around 5%, with values between 5.12 ± 0.12% and 5.64 ± 1.04%. However, at 175 °C and 200 °C, the breaking strain increased markedly to 7.33 ± 0.95% and 7.26 ± 0.51%, respectively. Although increased crystallinity is generally associated with reduced ductility, the results demonstrated that both strength and elongation increased at the highest hot-pressing temperatures. This behavior suggests that, in addition to beta phase growth, elevated hot-pressing temperatures improved interlayer bonding and reduced internal defects generated during the 3D printing process. The improved structural integrity allowed the material to withstand larger deformation before fracture [38]. Also, the highly oriented β-phase structure formed supports the load better, and the chains are well aligned in the plane direction, allowing for relatively large elastic deformation, making it possible to secure strength and flexibility simultaneously [39]. The toughness results reflected this combined enhancement. While the raw and lower-temperature samples exhibited toughness values around 0.09 to 0.10 J, the toughness increased significantly to 0.14 ± 0.04 J at 175 °C and 0.16 ± 0.03 J at 200 °C. The increase in energy absorption capability resulted from the simultaneous improvement in tensile strength and elongation. The fracture surface images in Table 3 show that brittle cracking features are more apparent in the raw and low-temperature samples, where irregular and fragmented fracture patterns are observed. In contrast, the specimens hot-pressed at 150–200 °C exhibit smoother and cleaner fracture surfaces with reduced crack fragmentation, indicating a more stable and cohesive fracture behavior at higher hot-pressing temperatures. Overall, increasing the hot-pressing temperature promoted β-phase development and crystalline growth, which led to enhanced stiffness and tensile strength. At 175 and 200 °C, additional improvements in structural integrity contributed to increased elongation, resulting in a substantial increase in toughness. Therefore, hot-pressing at the higher temperature range provided the most balanced mechanical performance in the 3DP_PVDF structures.

### 3.7. Electrical Properties of 3DP_PVDF_HP Samples

Figure 5c shows the electrical properties of 3DP_PVDF_HP with an applied voltage ranging from 0 to 50 V. All samples displayed a similar I-V trend, where the current gradually increased with increasing voltage. The electrical response of PVDF is strongly governed by its electroactive β-phase, which provides aligned dipoles responsible for its piezoelectric behavior. Previous studies have demonstrated that enhancement of β-phase content in PVDF-based systems directly improves electrical output and energy harvesting performance [40]. The current of all samples was measured in the range of −2 × 10^−7^ to 3 × 10^−7^ mA over an applied voltage range of −1 to 50 V. For better comparison of the electrical behavior of different 3DP_PVDF_HP samples, the current response was normalized. Similar normalization principles have also been widely adopted in PVDF-based electrical and electromechanical studies to suppress baseline variations and improve comparability of electrical signals [41]. This normalization minimizes the influence of initial current differences and enables reliable comparison of electrical performance [42].

As shown in Figure 5c, the normalized current increases with applied voltage for all samples. Over the voltage range of 0–50 V, clear differences were observed with hot-pressing temperature. The normalized current increases by approximately 96% for the sample hot-pressed at 100 °C, 142% at 125 °C, 178% at 150 °C, 340% at 175 °C, and 196% at 200 °C. Samples hot-pressed at 175 °C and 200 °C exhibited higher normalized current responses than those pressed at 100 °C and 125 °C. This behavior suggests that higher hot-pressing temperatures promoted more effective formation and continuity of conductive pathways within the PVDF structure, leading to enhanced charge transport under an applied electric field, in agreement with voltage-dependent conduction and percolation-based interpretation [43].

## 4. Conclusions

This study aimed to combine the use of 3D printing and hot-pressing as a process to enhance the surface quality, structural uniformity, and crystallinity of PVDF. By controlling the printing and hot-pressing conditions, the internal structure of the material was improved. As a result, the mechanical and electrical performance of PVDF was enhanced, indicating its potential suitability for piezoresistive applications, electromechanical devices, flexible sensors, and energy-harvesting systems.

Thermal analysis confirmed stable melting behavior and sufficient thermal stability for 3D printing. Rheological measurements showed low tan δ values below 130 °C and an increase above 150 °C, reflecting a transition from solid-like to flow-dominated behavior. DSC and TGA showed stable thermal transitions and degradation temperatures well above the processing range for safe printing conditions. Based on these findings, extrusion tests were performed at nozzle temperatures between 220 and 260 °C. Unstable flow was observed at 220–230 °C, and surface defects appeared at 240 °C, whereas smooth and uniform extrusion was obtained at 250 °C. Although PVDF remained thermally stable at 260 °C, slight dimensional instability was observed. Therefore, 250 °C was selected as the optimal printing temperature. After printing, hot-pressing at temperatures between 100 °C and 200 °C produced a gradual reduction in thickness without weight loss, demonstrating effective densification and improved surface smoothness. FTIR and XRD analyses showed retention of the dominant electroactive β-phase, along with enhanced crystalline ordering after hot-pressing. DSC analysis showed similar melting temperatures with smoother peaks and increased melting enthalpy at moderate hot-pressing temperatures, with improved thermal uniformity. Tensile property analysis showed that samples hot-pressed at 150 °C and 175 °C exhibited the most favorable balance between strength and flexibility. Electrical measurements showed stable voltage-dependent behavior for all samples. Samples hot-pressed at 175 °C and 200 °C exhibited higher normalized current responses than those processed at lower temperatures, corresponding to improved electrical connectivity and more continuous conductive pathways within the PVDF structure.

These results demonstrated that careful control of printing temperature and hot-pressing conditions preserved the electroactive β-phase while enhancing crystallinity and functional performance. The combination of optimized 3D printing and moderate hot-pressing provided a reliable approach for improving the thermal, mechanical, and electrical properties of PVDF. Future studies will examine composite structures based on PVDF and carbon materials fabricated using dual-nozzle FDM 3D printing. Alternative printing orientations, optimized pressure conditions, and additional post-processing strategies aimed at further improving structural densification and electrical continuity will be investigated. These approaches are expected to further enhance the structure-property relationship and electrical response of PVDF-based systems for sensing and energy-related applications.

## Figures and Tables

**Table 1:** Extrusion test of PVDF filament.

Nozzle Temperature (°C)	Thickness (mm)	Weight (mg)	Morphology
Raw	2.47 ± 0.02	147 ± 0.55	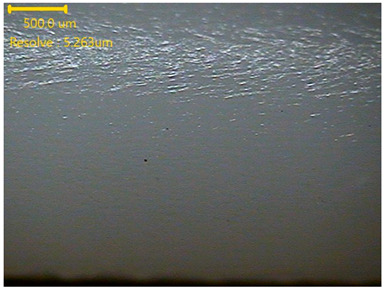
220°	0.53 ± 0.02	2.70 ± 0.01	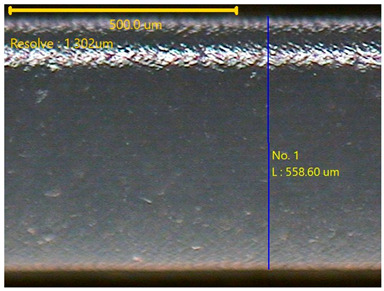
230°	0.52 ± 0.01	2.69 ± 0.01	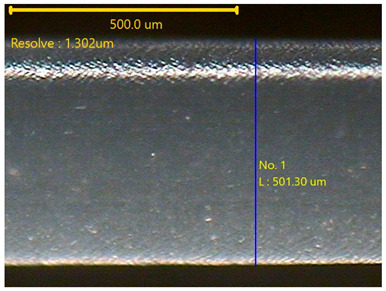
240°	0.59 ± 0.02	2.89 ± 0.01	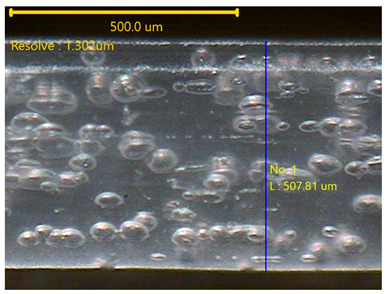
250°	0.60 ± 0.01	2.90 ± 0.01	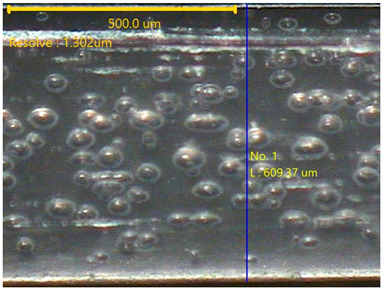
260°	0.54 ± 0.02	2.90 ± 0.01	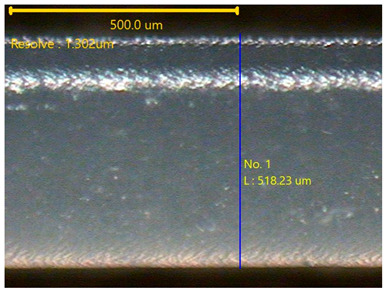

**Table 2:** Morphology and specification of 3DP_PVDF_HP samples.

Sample	Weight (g)	Thickness (mm)	Ratio of Decreased Thickness (%)	Sample Images	Morphology	
					Front	Back
PVDF	0.26 ± 0.00	0.25 ± 0.00	-	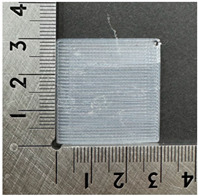	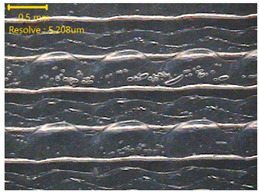	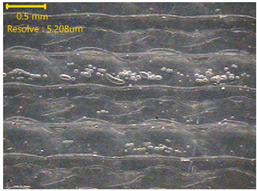
3DP_PVDF_HP100	0.26 ± 0.00	0.22 ± 0.00	10.67 ± 2.31	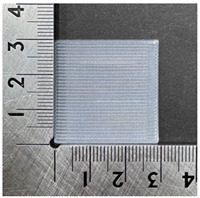	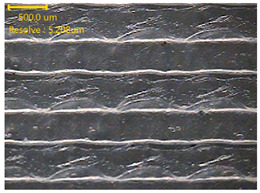	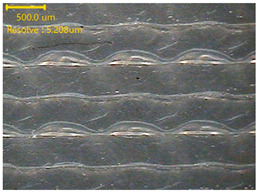
3DP_PVDF_HP125	0.26 ± 0.00	0.22 ± 0.00	12.00 ± 0.00	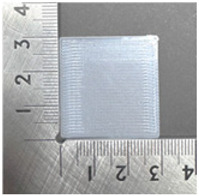	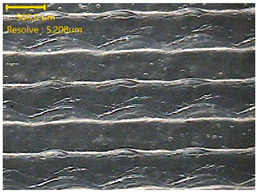	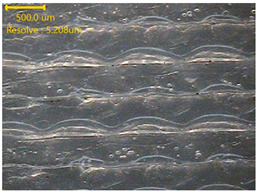
3DP_PVDF_HP150	0.26 ± 0.00	0.21 ± 0.01	14.67 ± 2.31	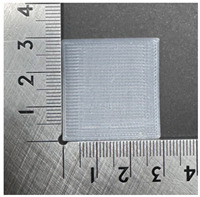	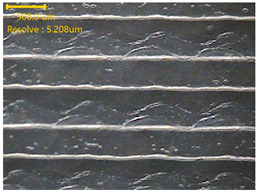	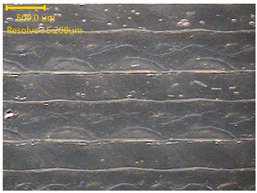
3DP_PVDF_HP175	0.26 ± 0.00	0.21 ± 0.00	16.00 ± 0.00	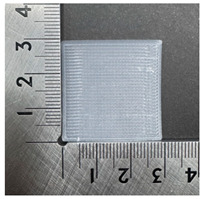	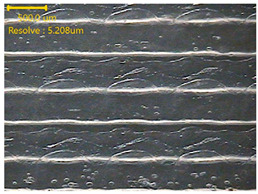	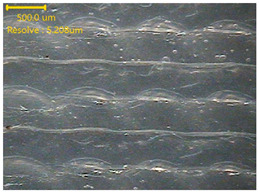
3DP_PVDF_HP200	0.26 ± 0.00	0.19 ± 0.00	24.00 ± 0.00	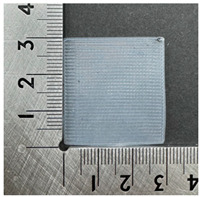	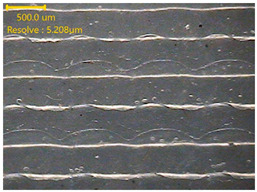	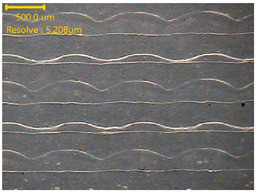

**Table 3:** Tensile property of 3DP_PVDF_HP samples.

Hot-Pressing Temperature (°C)	Initial Modulus (MPa)	Stress_max_ (MPa)	Breaking Strain (%)	Toughness (J)	Fracture Surface
Raw	785.08 ± 04.84	18.37 ± 0.96	5.64 ± 1.04	0.09 ± 0.01	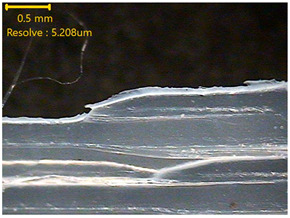
100°	937.67 ± 10.31	21.64 ± 0.55	5.18 ± 0.76	0.10 ± 0.01	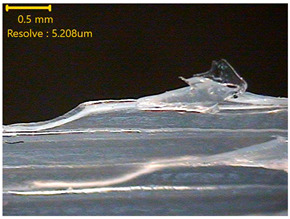
125°	945.70 ± 50.31	22.92 ± 0.97	5.15 ± 0.41	0.10 ± 0.01	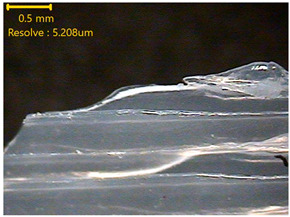
150°	979.45 ± 32.31	23.60 ± 0.74	5.12 ± 0.12	0.10 ± 0.01	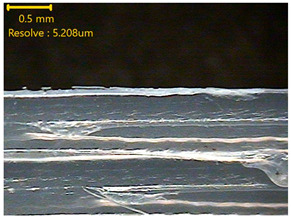
175°	988.94 ± 13.22	24.88 ± 1.97	7.33 ± 0.95	0.14 ± 0.04	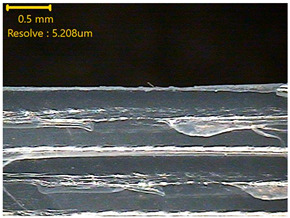
200°	1046.48 ± 116.36	28.72 ± 4.55	7.26 ± 0.51	0.16 ± 0.03	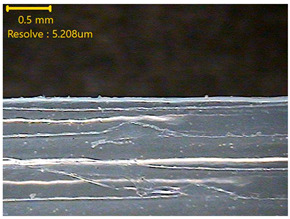

## Data Availability

The raw data supporting the conclusions of this article will be made available by the authors upon request.

## Abbreviations

The following abbreviations are used in this manuscript:

PVDF	Polyvinylidene fluoride
FDM	Fused Deposition Modeling
PTFE	Polytetrafluoroethylene
3DP_PVDF_HP	3D-printed PVDF hot-pressed sample
DSC	Differential scanning calorimetry
TGA	Thermogravimetric Analysis
FTIR	Fourier transform infrared
XRD	X-ray diffraction
